# NMR Study Reveals the Receiver Domain of Arabidopsis ETHYLENE RESPONSE1 Ethylene Receptor as an Atypical Type Response Regulator

**DOI:** 10.1371/journal.pone.0160598

**Published:** 2016-08-03

**Authors:** Yi-Lin Hung, Ingjye Jiang, Yi-Zong Lee, Chi-Kuang Wen, Shih-Che Sue

**Affiliations:** 1 Institute of Bioinformatics and Structural Biology, National Tsing Hua University, Hsinchu, Taiwan; 2 Instrumentation Center, National Tsing Hua University, Hsinchu, Taiwan; 3 National Key Laboratory of Plant Molecular Genetics, CAS Center for Excellence in Molecular Plant Sciences, Institute of Plant Physiology and Ecology, Shanghai Institutes for Biological Sciences, Chinese Academy of Sciences, Shanghai, China; 4 Department of Life Science, National Tsing Hua University, Hsinchu, Taiwan; Institute of Genetics and Developmental Biology, Chinese Academy of Sciences, CHINA

## Abstract

The gaseous plant hormone ethylene, recognized by plant ethylene receptors, plays a pivotal role in various aspects of plant growth and development. ETHYLENE RESPONSE1 (ETR1) is an ethylene receptor isolated from Arabidopsis and has a structure characteristic of prokaryotic two-component histidine kinase (HK) and receiver domain (RD), where the RD structurally resembles bacteria response regulators (RRs). The ETR1 HK domain has autophosphorylation activity, and little is known if the HK can transfer the phosphoryl group to the RD for receptor signaling. Unveiling the correlation of the receptor structure and phosphorylation status would advance the studies towards the underlying mechanisms of ETR1 receptor signaling. In this study, using the nuclear magnetic resonance technique, our data suggested that the ETR1-RD is monomeric in solution and the rigid structure of the RD prevents the conserved aspartate residue phosphorylation. Comparing the backbone dynamics with other RRs, we propose that backbone flexibility is critical to the RR phosphorylation. Besides the limited flexibility, ETR1-RD has a unique γ loop conformation of opposite orientation, which makes ETR1-RD unfavorable for phosphorylation. These two features explain why ETR1-RD cannot be phosphorylated and is classified as an atypical type RR. As a control, phosphorylation of the ETR1-RD was also impaired when the sequence was swapped to the fragment of the bacterial typical type RR, CheY. Here, we suggest a molecule insight that the ETR1-RD already exists as an active formation and executes its function through binding with the downstream factors without phosphorylation.

## Introduction

Ethylene (C_2_H_4_) is the first identified gaseous hormone in plants and is involved in various aspects of plant growth and development [1–3]. The Arabidopsis genome encodes five ethylene receptor isoforms that are classified into two subfamilies depending on their primary sequences and domains [4]. The subfamily I members ETHYLENE RESPONSE1 (ETR1) and ETHYLENE RESPONSE SENSOR1 (ERS1) have the signature motifs of histidine kinase (HK) domains. The signature motifs are largely lacking for the subfamily II members ETR2, ETHYLENE INSENSITIVE4 (EIN4) and ERS2, where the three members have *in vitro* Ser/Thr kinase activity [3]. Moreover, the subfamily I and II family members have three and four putative transmembrane helices, respectively [5].

ETR1 is the first identified ethylene receptor protein and becomes the most studied [6]. ETR1 N-terminal transmembrane helices form a hydrophobic pocket for ethylene binding, requiring the cuprous ion (Cu^+^) as a cofactor [5, 7]. With ethylene binding, the receptors are inactivated and cannot mediate receptor signaling to repress ethylene signaling. Following the ethylene-binding helices, there are the cyclic GMP-regulated phosphodiesterase (GAF) and HK domain. The HK domain is the docking site of the Raf-like CONSTITUTIVE TRIPLE-RESPONSE1 (CTR1) protein that mediates the receptor signal output to repress ethylene signaling [8, 9]. The GAF domain has a role in inter-receptor interaction and may mediate receptor signaling via an alternative pathway that is independent of CTR1 [10]. The receiver domain (RD) following the HK domain is present in the ETR1, ETR2 and EIN4 receptors. Little is known about the exact role of the RD in ethylene signaling.

**Fig 1:**
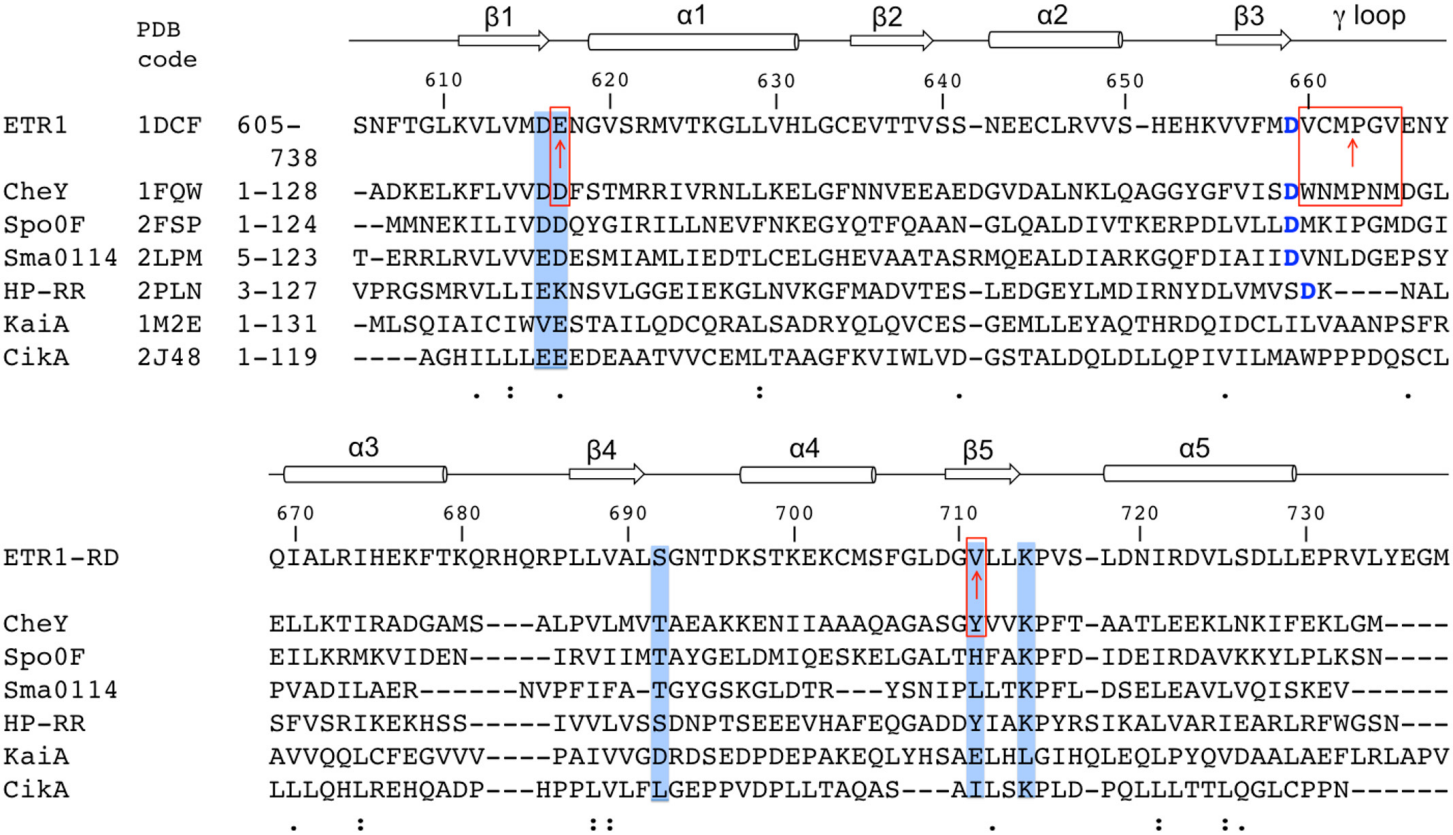
Multiple alignments of the various available receiver domain structures. The secondary structure elements of the ETR1-RD are shown above the alignments, with arrows representing the β-strands and cylinders representing the α-helixes. The residues critical for phosphorylation and metal binding are shown with a blue-shaded background, and the residues mutated to match the CheY sequence are indicated by red boxes. The conserved aspartate is indicated by blue font. The alignment was generated using Clustal Omega with manual adjustment.

The ETR1 has been linked to a prokaryotic two-component system (TCS) because the C-terminal RD sequence is similar to the components of the signal transducers identified in the prokaryotic TCS [6]. The TCS has been widely found in transcriptional regulatory networks, involving phosphorelay [11, 12]. A typical TCS consists a membrane-bound histidine kinase (HK) module for signal sensing and a corresponding receiver regulator (RR) as the signal output transducer, in which the autophosphorylated histidine residue transfers the phosphoryl group to the aspartate residue of the RR to induce corresponding responses [13]. The ETR1 HK domain structurally resembles the HK module, and the receptor has a RD at its C-terminus. The ETR1-RD, with the conserved aspartate residue, is structural homologous of the prokaryotic RRs, comprising a conserved structural fold of five antiparallel β-α repeats (Fig 1) [14, 15]. Therefore, the ETR1-RD has been believed to be able to receive the phosphoryl group delivered from the HK domain, and the ethylene signaling could be mediated through the phosphorelay scheme. However, evidence for the presence of ETR1-RD phosphorylation is lacking.

As a putative TCS, there is a controversy for the function of ETR1-HK and RD domains, and lines of evidences do not support the requirement of the two domains for ETR1 ethylene signaling. ETR1 ethylene signaling is not prevented when the RD domain or both of the HK and RD domains are removed [16]. An *in vitro* assay indicated that ETR1 has HK activity, but the kinase activity are not necessarily associated with receptor signaling because the impaired kinase activity did not affect the ethylene signaling [17]. Interestingly, signaling of the kinase-dead ETR1 receptor was stronger than that of its wild-type counterpart [18]. The ETR1-RD is also not needed for ETR1 in mediating the response from ethylene antagonist, silver [19]. Ethylene-treated etiolated Arabidopsis seedlings quickly resume growth upon ethylene removal, and the ETR1-RD without phosphorylation may have a role in growth recovery after ethylene treatment [18]. The Asp659 residue is the putative phosphoryl-accepting site of ETR1-RD, and expression of Asp-lacking *getr1-[D]* transgene could still in part restore the growth recovery of the *etr1-6;etr2-3;ein4-4* loss-of-function mutant, indicating that Asp659 is not necessary for growth recovery; the partial recovery was possibly due to the lesser expression of *getr1-[D]* [18]. On the other hand, *getr1-[D]* has same effects as the wild-type *ETR1* on restoring the growth of the non-treated *etr1-6;etr2-3;ein4-4* mutant [18]. Etiolated Arabidopsis seedlings nutate, stimulated by ethylene, after germination requires ETR1. Expression of *getr1-[D]* greatly rescued the nutation-defective phenotype of *etr1-6;etr2-3;ein4-4* mutant, also implying the less correlation between Asp659 and ethylene stimulation [20]. Moreover, whether phosphorelay is involved in the ETR1 receptor signal output was evaluated with the use of the *ARABIDOPSIS RESPONSE REGULATOR6* promoter to drive a luciferase reporter. ETR1^D659E^, with its putative phosphoryl group-accepting residue aspartate replaced with glutamate, induced similar reporter activity as the native ETR1, whereas the luciferase induction declined with the expression of the kinase-dead ETR1^H353Q, D659A^ [16]. Those results suggest the association of receptor signaling with HK activity but not with the RD phosphorylation status. Nevertheless, biochemical evidence is still lacking to address if ETR1-RD phosphorylation would occur and the RD function and its phosphorylation status is associated.

Bacterial RRs were classified into typical, pseudo, and atypical types, based on their sequences and phosphorylation status. Although ETR1-RD and bacterial RRs are structurally homologous, the type of ETR1-RD is to be determined. The differences between the three types of RRs are summarized here. The typical RRs contain a conserved aspartate residue at the end of β3 and accept a phosphoryl group from the phosphorylated histidine residue of the HK domain; examples are CheY, NtrC, PmrA, and Spo0F [21–25]. RRs that lack the conserved aspartate residue, such as CikA and KaiA, cannot be phosphorylated and belong to the pseudo type RRs [26, 27]. The pseudo type RRs have similar structures as the typical RRs and may function via binding with downstream molecules. There are a few RRs that are classified as the atypical type, containing the conserved phosphoryl-related aspartate residue that however cannot be phosphorylated. The HP-RRr is an example of the atypical type [28]. Fig 1 shows the sequence alignments of the ETR1-RD and common RRs. Based on the current knowledge about RRs, distinguishing typical and atypical RRs cannot be simply achieved by sequence comparison, and the type of the ETR1-RD is still unknown. Determining if the ETR1-RD phosphorylation would occur advances our knowledge about ETR1 receptor signaling.

Structural biology provides information about the macromolecular structure at the atomic level to explain how a molecule may acquire a function upon structural alteration. Studies of the structure of the ETR1-RD will help addressing the structural mechanisms underlying RD phosphorylation and receptor signaling. An X-ray diffraction study for a bacterially expressed ETR1-RD revealed that two ETR1-RDs constituted a structural unit in the crystal packing [29], providing a precise atomic structure of the protein, but it only represented a snapshot of an energy-favorable protein structure. The nuclear magnetic resonance (NMR) technique has been used for a similar purpose and is performed in solution to study the protein structure and dynamics under physiological conditions, providing a further characterization of protein motion and fluctuation that complements the information from the X-ray structure. In this study, we report the use of NMR to provide structural and dynamic information about the ETR1-RD in solution to advance our knowledge about the functions of the ETR1-RD. The solved X-ray structure was revealed to be a dimer, with an unusual dimer interface that the C-terminus only contained a short β-strand extending to another ETR1-RD. Our results firstly corrected that the ETR1-RD is monomeric in solution and we reported the solution structure. Secondly, we demonstrated the deficiency of the ETR1-RD for accepting the phosphoryl group. ETR1-RD is unable to bind the phosphate analog BeF_3_^-^ in solution, where BeF_3_^-^ treatment has been widely used to mimic the phosphorylation of the bacterial RRs [22, 30–33]. Thus, the ETR1-RD cannot be phosphorylated from a donor. The dynamics and structure of the ETR1-RD were compared with other bacterial RRs and the results provided mechanistic explanations for why the ETR1-RD cannot be phosphorylated. We conclude that the ETR1-RD can be classified as the atypical type. The possible functional significance of the RD in ETR1 receptor signaling is discussed.

## Results

### Structure of the ETR1-RD in solution

**Fig 2:**
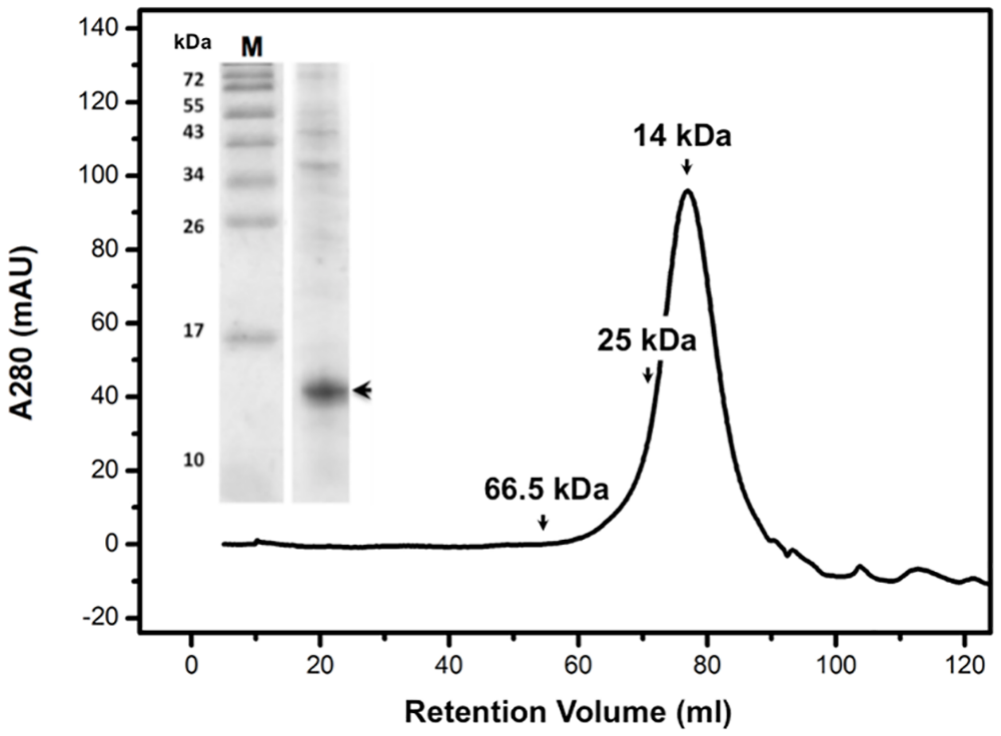
The FPLC size-exclusion elution profile of the ETR1-RD. The insert shows the purity and molecular weight of the ETR1-RD after Ni-column purification. The result indicates that the ETR1-RD is a monomer in solution.

The structure of the ETR1-RD has been previously determined by X-ray crystallography, and the solved structure was revealed to be a dimer, with the C-terminus forming a short β-strand extending to another ETR1-RD unit [29]. Fewer studies related to the properties of the ETR1-RD in solution have been performed. In addition to an X-ray diffraction study, the oligomerization status of the ETR1-RD in solution has been preliminarily investigated by NMR, and the results suggested that the RD was a monomer in solution [34]. Here, SDS-PAGE and FPLC analysis revealed that the ETR1-RD had a molecular weight of ~15 kDa in solution, further supporting the conclusion that it existed as a monomer in solution (Fig 2). These conflicting results prompted our study of the structure of the ETR1-RD in solution.

**Fig 3:**
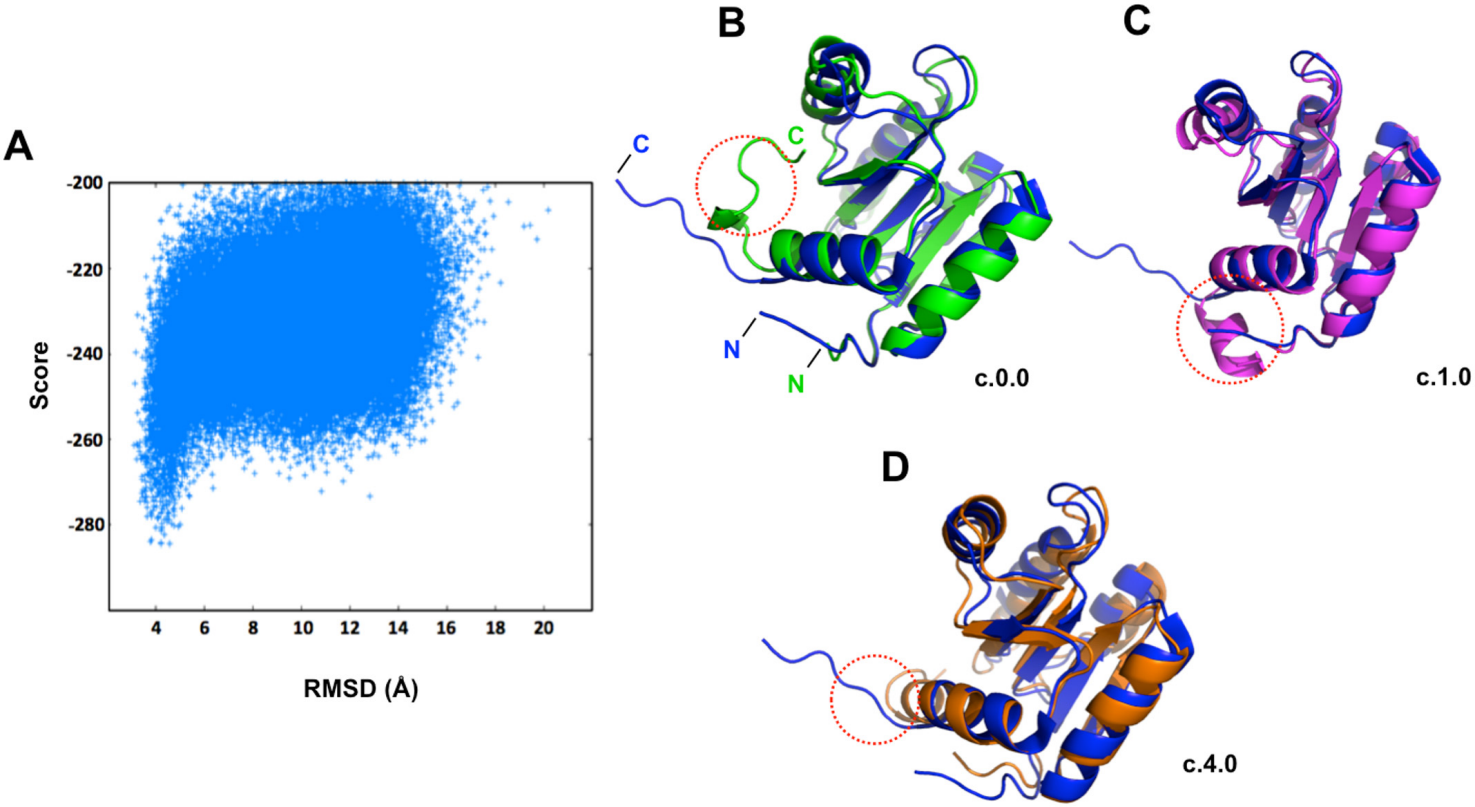
Structural determination of the ETR1-RD by Rosetta. (A) Plots of the Rosetta all-atom score versus Cα RMSD relative to the X-ray crystal structure (PDB code 1DCF). Representative structures of (B) the C.0.0 cluster (green), (C) the C.1.0 cluster (magenta) and (D) the C.4.0 cluster (orange) and the X-ray crystal structure (blue). The orange circles indicate the C-terminal structural difference in the structural modeling.

The resonances in the ETR1-RD ^1^H-^15^N HSQC spectrum are even and highly intense. The distribution of the resonances is dispersed throughout the spectrum. These properties indicate that the ETR1-RD is a highly folded domain with high conformational stability. The assignment was finished by adopting conventional backbone assignment strategy [34]. Based on this strategy, we were able to obtain the chemical shifts for all of the backbone nuclei. The values of the backbone chemical shifts are significantly affected by the protein backbone dihedral angles; therefore, they are sensitive to the protein’s secondary structure. For example, a secondary structural tendency could be established from the parameter (ΔCα−ΔCβ) where ΔCα and ΔCβ respectively represent the chemical shift differences between the protein Cα and Cβ chemical shifts and the corresponding values derived from a random coil. The (ΔCα−ΔCβ) profile of the ETR1-RD evidenced a very similar secondary structural pattern to that determined by X-ray crystallography [29], except that the C-terminal short β-strand was missing in the NMR estimation. To more precisely evaluate the differences between the solution and crystal structures, we used the protein structure modeling software, Rosetta, to calculate the ETR1-RD solution structure [35]. Rosetta has been shown to predict the structures of small proteins (< 150 residues) very accurately. We further incorporated the NMR backbone chemical shift information, including the chemical shifts of HN, Cα, Cβ, CO, Hα and Hβ, into the Rosetta model. Because backbone chemical shifts reflect secondary structural tendencies, the integration such chemical shifts critically improves the accuracy of the structure modeling. We generated 50,000 Rosetta structures. The plots of the Rosetta all-atom score were funneled with respected to Cα RMSD, while the RD crystal structure was referenced (Fig 3A). The distribution indicates the good structural convergence of the prediction. Based on structural similarity, we clustered the structures with the lowest RMSD values (S1 Table). Three major clusters containing the highest number of structures (124, 118 and 100 structures, respectively) were selected to represent the optimal RD models. The three clusters showed the greatest structural similarity to the X-ray structure, adopting the structural fold of a parallel five-stranded β-sheet core surrounded by five helices (Fig 3B–3D). A structural ambiguity in the prediction indicated that the C-terminal region adopted either a short helix (Fig 3B and 3C) or random coil structure (Fig 3D). All conformations have comparably low Rosetta scores, and it is impossible to determine the correct conformation by model analysis alone. We resolved the problem by studying the protein backbone dynamics.

### NMR backbone ^15^N relaxation data

**Fig 4:**
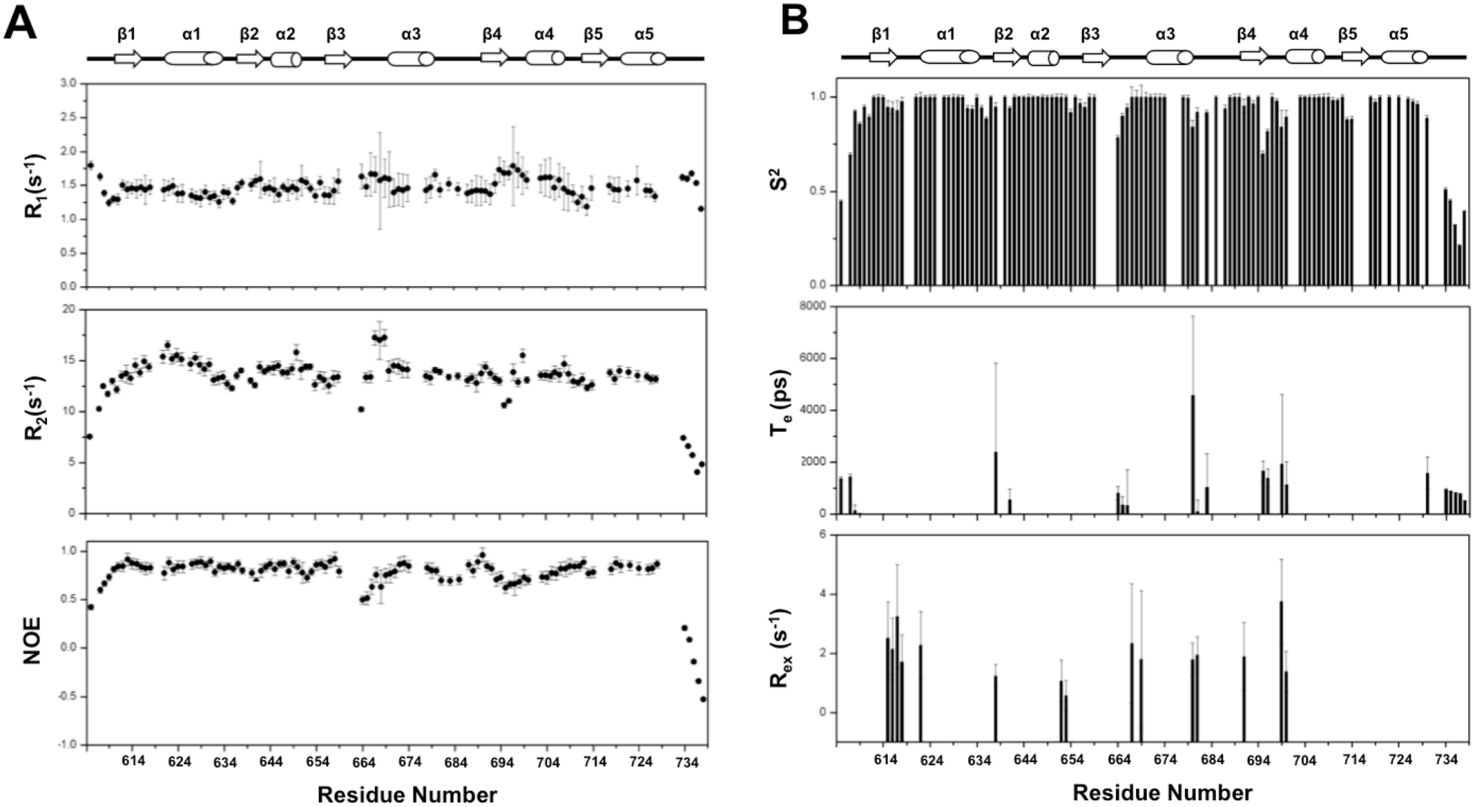
NMR dynamics information for the ETR1-RD backbone NH. (A) ^15^N relaxation parameters (R_1_, R_2_, NOE). The perturbations of the R_1_, R_2_, and NOE values are mostly observed in the loops and the terminal regions. (B) A model-free analysis based on the ^15^N relaxation parameters. Residues with high order parameters (S^2^ > 0.9) indicate structural rigidity. Residues with an observable T_e_ exhibit fast motion (ps) in solution, whereas residues with a R_ex_ perform a conformation exchange on a μs to ms time scale.

The NMR backbone amide ^15^N relaxation data for R_1_, R_2_, and the [^1^H]-^15^N heteronuclear NOE, were recorded at 600.13 MHz (Fig 4A). The R_1_ and R_2_ values of ^15^N indicate how fast the excited magnetization can “relax” back to a resting state. The relaxation rates are related to entire protein tumbling (on a ns time scale) and the backbone local fluctuation (< ns time scale). The [^1^H]-^15^N heteronuclear NOE of an individual N-H bond could be obtained by comparing the peak intensities in the particular experimental set with and without the proton saturation pulses [36]. The NOE value is sensitive to a motion that is faster than the overall tumbling of the molecule. The R_1_, R_2_ and [^1^H]-^15^N NOE values together indicate protein backbone motion on a ps-ns time scale.

We analyzed the values of the relaxation data for 114 NH resonances of the ETR1-RD after excluding the overlapping resonances and the proline residues (proline has no NH). For the residues located in the secondary structure, the rates are distributed around the average value, indicating general structural rigidity. Meanwhile, the increased R_1_ and reduced R_2_ and NOE occur on the flexible regions, representing structural flexibility. It is particularly interesting in the ETR1-RD that the C-terminal region following α5 has very low NOE values, indicating dynamic properties (Fig 4A). The C-terminal region is flexible, containing no secondary structure. This result supports a random coil structure, as shown in Fig 3D. Thus, the predicted short α-helix appearing in Fig 3B and 3C and the β-strand in the X-ray crystal structure do not represent the correct C-terminal structure in solution.

The overall molecular tumbling time (rotational correlation time, τ_c_) is obtained from the average ratio of R_1_ and R_2_ and is estimated to be 8.6 ns, corresponding to a molecular weight lower than 20 kDa. This confirms again that the ETR1-RD is monomeric, leading to the conclusion that the dimeric interface observed in the X-ray structure is due to crystal packing. Our study demonstrated that X-ray diffraction provides a precise atomic structure of the ETR1-RD, but contrarily, FPLC size-exclusion chromatography and an NMR relaxation experiment indicate that the ETR1-RD is a monomer in solution. The difference might not be derived from the buffer condition that HEPES buffer at neutral pH was used in X-ray crystallization and Tris buffer was for NMR study. Both buffers did not convert ETR1-RD into a dimer in the NMR measurement. A recent study involving small angle X-ray scattering (SAXS) also suggested the RD was not dimerized [37]. The SAXS modeling of the bacterially expressed ETR1^158-738^, a soluble fragment that lacks the transmembrane helices, indicates that the HK domain is dimerized, and the RD are separate from each other. Moreover, unlike the X-ray diffraction study that predicted the C-terminus to be a short β strand, our NMR relaxation result suggests that the C-terminal end is unrestricted and behaves like a random coil.

### Model-free analysis of ^15^N relaxation data

In addition to the direct observation of R_1_, R_2_, and NOE, we quantified the dynamics by model-free analysis using the Tensor2 program (Fig 4B). Three dynamic parameters of S^2^, T_e_ and R_ex_ were extracted that respectively represent the order parameter, backbone fluctuation (< ns time scale) and conformational exchange (μs-ms time scale). The high S^2^ values (> 0.9) indicate backbone rigidity. The N- and C-terminal regions and the loops contain regional fluctuations with T_e_ values and reduced S^2^. In additional, we noticed ~15 residues exhibiting R_ex_. These residues might undergo a conformational exchange on a μs/ms time scale. These residues are sparsely distributed in the ETR1-RD structure. There is no large-scale collective motion.

### Phosphorylation of the ETR1-RD

**Fig 5:**
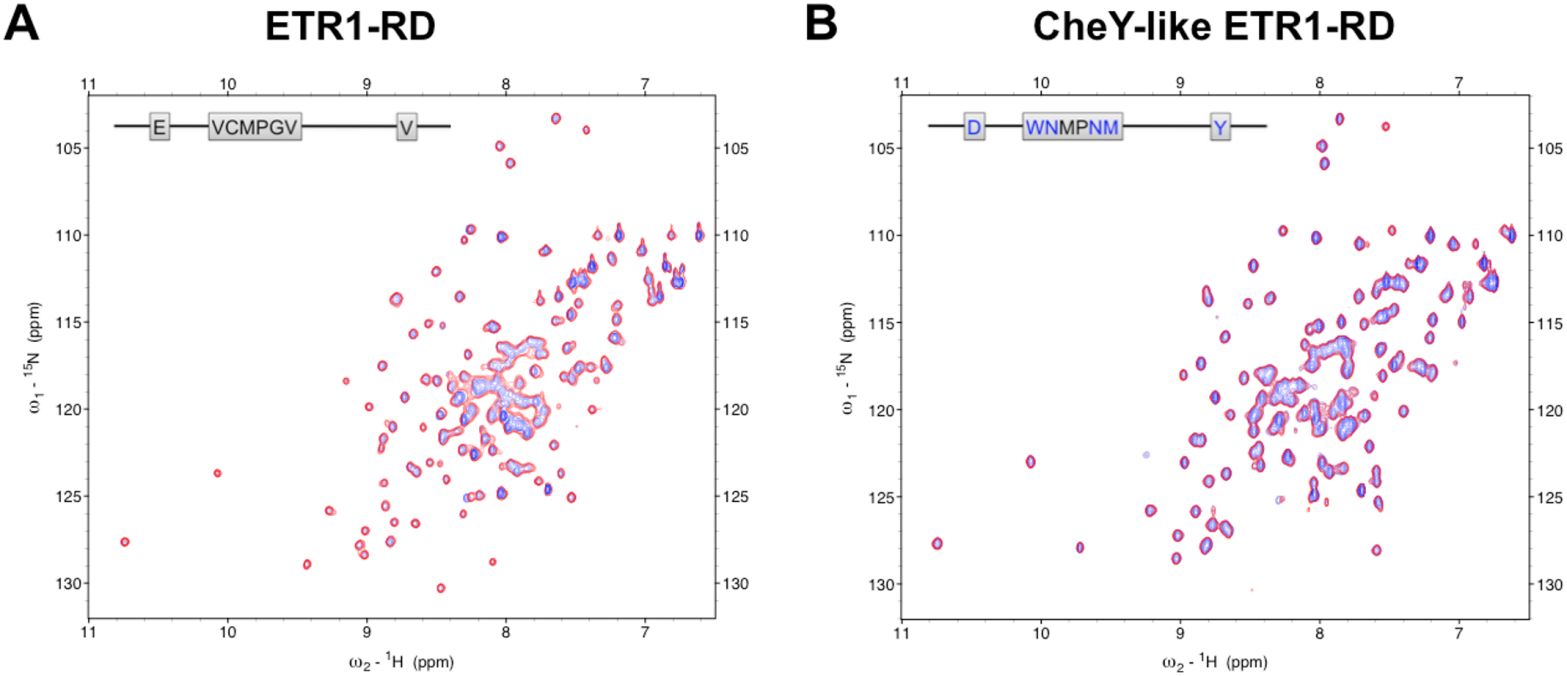
HSQC spectrums of BeF_3_- titration. HSQC titrations of (A) the ETR1-RD and (B) the CheY-like ETR1-RD. ^1^H-^15^N HSQC of RD samples with ~ 0.1 mM protein in NMR buffer. The spectra of the free proteins are blue. The spectra of proteins titrated with 3 mM BeF_3_^-^/10 mM Mg^2+^ are red. The inserts depict the sequences of the ETR1-RD and the CheY-like ETR1-RD.

The phosphorylation of the conserved aspartate residue of the RD (or RR) serves as a critical structural intermediate in the phosphoryl-transfer scheme of the TCSs. A phosphorylated RD acts like an active conformer to either transfer the phosphoryl group to the next molecule or initiate the downstream signaling through a protein-protein interaction. We evaluated whether the ETR1-RD could be phosphorylated by titrating with beryllofluoride (BeF_3_^-^). Because of the short half-life of aspartate phosphorylation, BeF_3_^-^ has been generally used to mimic the phosphorylation of the RD. An typical type of bacterial RR that can be phosphorylated can form a stable BeF_3_^**—**^Asp linkage [38]. Thus, the structural intermediate has been treated as an aspartyl phosphate analog. A common procedure is the titration of BeF_3_^-^ into an RD (or RR) solution [39, 40]. Additionally, divalent cations, usually Mg^2+^, Mn^2+^ or Ca^2+^, are required for the phosphorylation of the aspartate residue. When using the phosphate analog BeF_3_^-^, the conserved active site also binds the divalent metals. In our NMR study, we titrated Mg^2+^ and BeF_3_^-^ into an ETR1-RD solution. A comparison of the HSQC spectra of the ETR1-RD alone and the ETR1-RD with Mg^2+^•BeF_3_^-^ revealed that the amide resonances showed no perturbations (Fig 5A), indicating that the ETR1-RD is insensitive to Mg^2+^ and Mg^2+^•BeF_3_^-^. Surprisingly, the ETR1-RD is not a typical type RD.

### Phosphorylation of CheY and CheY-like ETR1-RD

**Fig 6:**
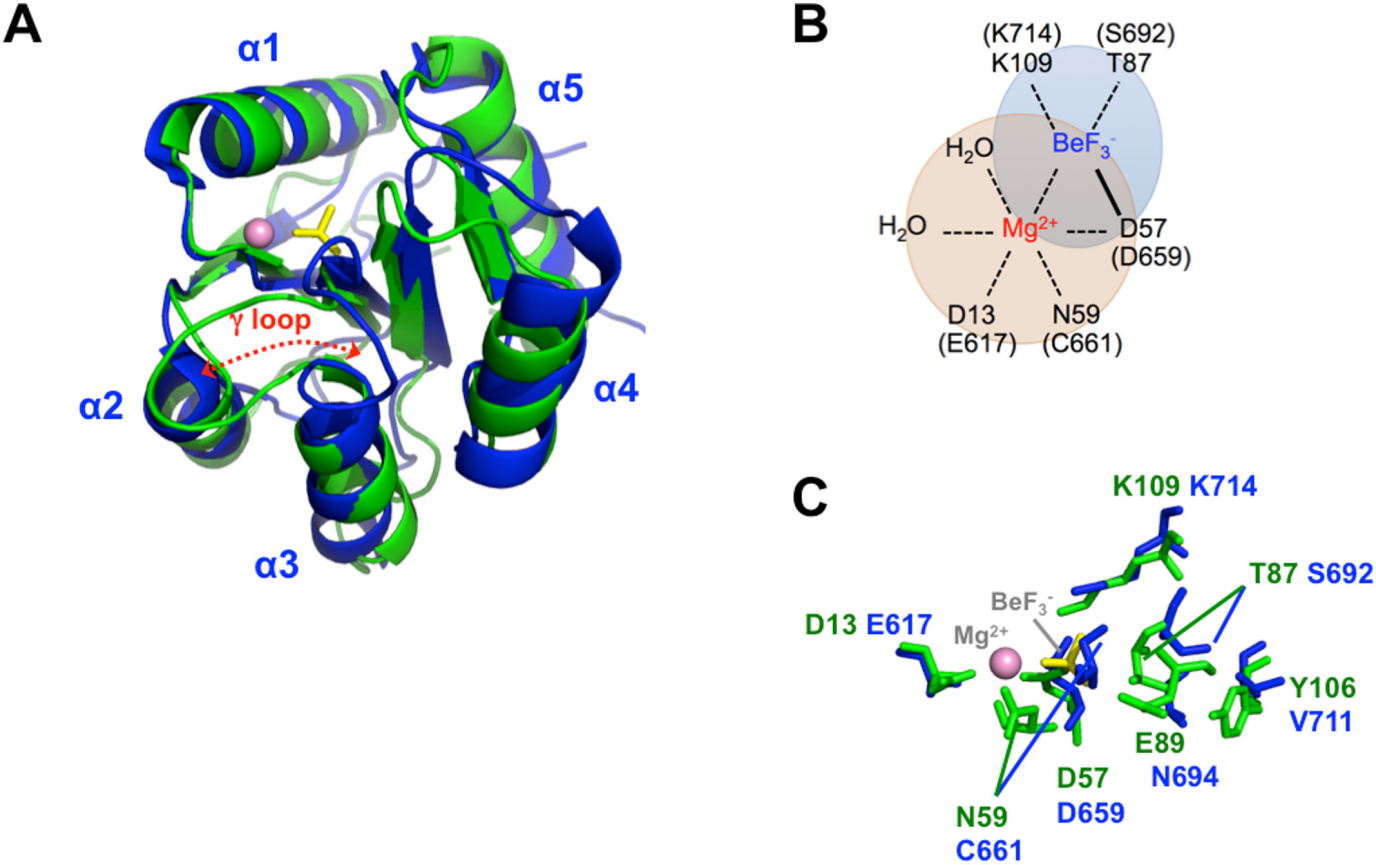
The ETR1-RD structure and a comparison with CheY. (A) Superimposed ETR1-RD (blue) and phosphorylated CheY (green, PDB code 1FQW). The Mn^2+^ metal and BeF_3_^-^ conjugated in the CheY structure are shown as a pink sphere and a yellow stick, respectively. (B) The coordination geometry in the vicinity of Mn^2+^ and BeF_3_^-^ in CheY. The corresponding residues of ETR1-RD are indicated in parentheses. (C) The spatial distributions of the residues surrounding the phosphorylation site.

Although the ETR1-RD has sequence similarity with typical RRs, the sequence differences might be responsible for the binding deficiency. We tried to restore the Mg^2+^•BeF_3_^-^ binding activity by changing the sequence. CheY has been widely studied because of its typical response regulator properties [21, 41]. The structural comparison is shown in Fig 6. Using the typical CheY-RR as a template, CheY Asp57, the conserved phosphorylation site on the β1-α1 loop, binds BeF_3_^-^, and the Thr87 and Lys109 side chains form hydrogen bonds and a salt-bridge with BeF_3_^-^, respectively (Fig 6B). The six coordination sites of the metal are filled by the side chain oxygens of Asp13 and Asp57, the carbonyl oxygen of Asn59, the fluorine of BeF_3_^-^, and two water molecules (Fig 6B). In the ETR1-RD, the corresponding residues are Asp659, Ser692 and Lys714 for BeF_3_^-^ binding and Glu617, Asp659, Cys661 for metal binding (Figs 1 and 6C). Based on this comparison, we mutated the ETR1-RD Glu617 to Asp. Because the ETR1-RD β3-α3 loop (γ loop) has a different conformation (Fig 6A), the Cys661 adopts an unfavorable orientation when binding with metal. We substituted the entire loop sequence (Val660 to Val665) with the corresponding sequence of CheY (Trp58 to Met63). Finally, because CheY Tyr106 has been assumed to crucially stabilize the orientation of Thr87 by a mechanism defined as Y-T coupling [22], we also substituted the corresponding residue Val711 with Tyr. Overall, the Glu617 and Val711 residues of CheY were respectively substituted with Asp and Tyr, replacing six residues on β3-α3 loop to create a CheY-like ETR1-RD (as shown by the red boxes in Fig 1). The HSQC of the CheY-like ETR1-RD variant is similar to the spectrum of the wide-type RD, indicating no significant structural changes in the mutation (Fig 5B). We compared the HSQCs in the absence and presence of Mg^2+^•BeF_3_^-^ to evaluate their phosphorylation status in the CheY-like ETR1-RD variant (Fig 5B), and consistently, the peaks showed no perturbation. The BeF_3_^-^ binding ability could not be restored by replacing the critical CheY residues in the ETR1-RD sequence.

### Backbone dynamics comparison of typical- and atypical-type response regulators

**Fig 7:**
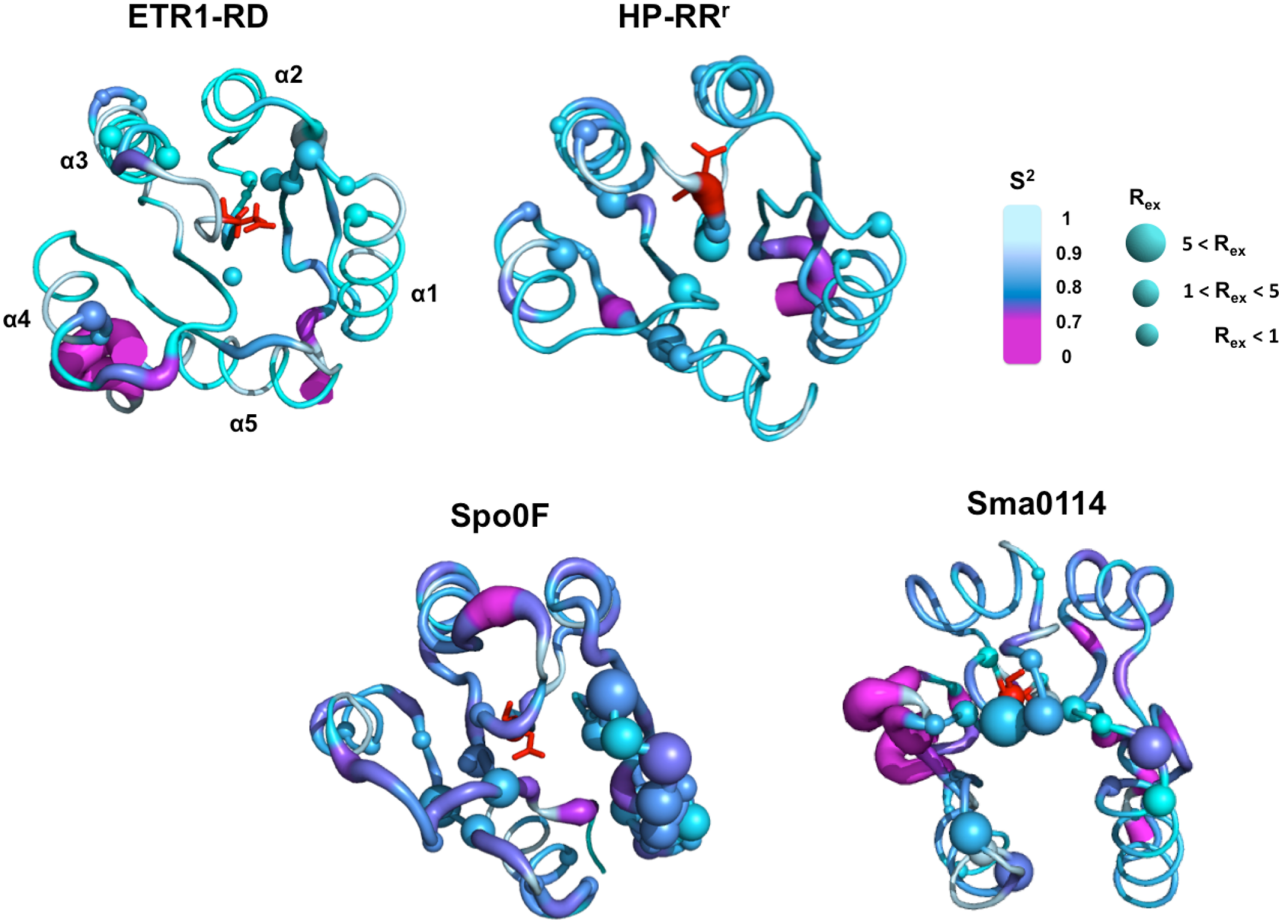
Comparison of the dynamic parameters for different RDs. The derived dynamic parameters S^2^ and R_ex_ of the ETR1-RD, HP-RRr (atypical type) [43] and Spo0F [42], Sma0114 (typical type) [41] are mapped onto their structures with colors ranging from magenta (flexible) to cyan (rigid). The residues depicted by balls indicate the presence of detectable R_ex_, the size reflecting the scales. The red sticks indicate the residues that correspond to the conserved aspartate site.

In the NMR Mg^2+^•BeF_3_^-^ titration experiment, phosphorylation of the ETR1-RD and the CheY-like ETR1-RD variant was not detected; the latter was substituted with the related-phosphorylation residues of the CheY sequence for the ETR1-RD. The CheY-like variant has no ability to interact with Mg^2+^ nor is phosphorylated, indicating that the backbone dynamics are as important as the structure for determining the ability to accept a phosphoryl group in the ETR1-RD. We mapped the dynamic parameters S^2^ and R_ex_ on the ETR1-RD structure and compared them with other RRs (Fig 7). In the ETR1-RD, although some residues surrounding the proposed phosphorylation site exhibit a conformational exchange, including Met615, Asp616, Glu617, Asn618 located on the β1-α1 loop, Asn667, Gln669 located at the beginning of α4, and Leu691 located on β4, the values of R_ex_ are relatively small. We did not see a large-scale collection motion in the ETR1-RD. We compared the dynamic parameters of other types of RDs: the HP-RR^r^ as the atypical type and, Spo0F and Sma0114 as typical types (Fig 7) [41–43]. The three structures contain no phosphoryl group nor bind to BeF_3_^-^. The apo Spo0F has generally smaller S^2^ values, indicating greater backbone flexibility (Fig 7) and shows many residues with a large-scale R_ex_. These residues are especially distributed on the structural segment of α1. Meanwhile, apo Sma0144 has greater backbone flexibility and a large-scale R_ex_ of the metal binding and phosphorylation site [41]. On the contrary, HP-RR^r^, behaved like the ETR1-RD, with a very small R_ex_ and high S^2^ values. Thus, if not phosphorylated, Spo0F and Sma0114 are dynamic. This is consistent with the observations that typical RRs are structurally flexible prior to phosphorylation. The incorporation of Mg^2+^ or other metals and BeF_3_^-^ stabilizes the structure and eliminates the backbone fluctuation [44]. However, the HP-RR^r^ and the ETR1-RD already have very limited backbone dynamics. These atypical RDs and RRs have less propensity for conformational exchange.

## Discussion

The major structural differences between the ETR1-RD and other typical RDs are located in the γ loop. Typical RDs have an γ loop near helix α2 and the γ loop in the ETR1-RD is flipped to the opposite side, interacting with helix α4 (Fig 6A) [29, 45]. This unique feature causes the critical residues for the phosphoryl-relay mechanism to point in different directions; specifically, the Asp659 side chain oxygen is hydrogen-bound with the Lys714 side chain, and Cys661 and Ser692 adopt an unfavorable orientation for binding a metal and phosphate, respectively [29]. This structural difference makes the occurrence of phosphorylation rather unlikely. Considering protein dynamics, typical RRs have been shown to contain residues that experience significant R_ex_. The R_ex_ clusters have been observed in the region surrounding the phosphorylation and metal-binding sites [41, 46] indicating an ability to allow structural interconversion between an inactive and active state [41, 47]. Backbone flexibility has been believed to be associated with binding to downstream targets subsequent to a ligand-induced conformational change, thereby modulating the binding specificity [47]. Thus, the dynamics reflect the ability of an apo RR or RD to assume to a phosphorylated state. Typical RDs such as Spo0F contain a dynamic helix α1 and Sma0114, EL LovR and CKl1-RD have flexible γ loops [41, 45–47] that Spo0F and Sma011 have a large-scale R_ex_ and relatively smaller S^2^ values surrounding the metal binding site and the phosphorylation region. In the case of sensor histidine kinase CKl1, Mg^2+^ binding stabilizes the γ loop and CKl1-RD in complex with Mg^2+^ is readily for subsequent phosphorylation [45]. If the γ loop of the ETR1-RD also has a flexible backbone, a ligand-induced conformational change might allow phosphorylation to occur. However, Mg^2+^ did not induce the structural rearrangement of the ETR1-RD, even at a very high concentration (>10 mM). The ETR1-RD structure is rigid and the dynamic properties are similar to the atypical HP-RR^r^. Due to its limited structural flexibility, we expect that no phosphorylation occurred. In addition, some RRs have been reported to form a homodimer after phosphorylation [24, 48]. We did not observe the ETR1-RD to form a dimer under any conditions, even in the presence of Mg^2+^ and/or the phosphoryl analog, BeF_3_^-^. That relative conformational rigidity and γ loop conformation difference are the factors that cause the phosphorylation deficiency of the ETR1-RD.

With the use of alanine-scanning mutagenesis of the ETR1-RD [19], the amino acid substitutions of ETR1-RD at either the phosphorylation site (D659A and C661A) or the metal binding site (E617A and C661A) did not impair the growth recovery trait and ethylene-stimulated nutation [19]. The growth recovery and nutation traits are independent of the RD phosphorylation. Moreover, RD-lacking ETR1 variants are still capable of ethylene signaling suppression [49–51]. Our structural biology study that suggested deficiency of phosphorylation of the ETR1-RD agrees with the receptor signaling being independent of its phosphorylation. Interestingly, the alanine-scanning study unexpectedly revealed three mutations at the RD, Q684A, E730A and L734A at the C-terminal tail, apart from the conserved Asp659 residue, impairing ETR1 receptor-mediated seedling nutation. Moreover, the RD-truncated *etr1-11*, resulting from the Q681Stop early termination, cannot convey ETR1 signaling output [52]. The C-terminal region might have a role in binding other proteins for different receptor signaling. However, this scenario is not supported for the RD-lacking ETR1 being capable of ethylene signaling suppression. Besides, the HK domain also appears to have a role in receptor signal output. The *etr1-5* and *etr1-8* mutations, resulting from the Trp563Stop early termination at the HK domain, and the *etr1-13* mutation, resulting from the G560D substitution at the HK domain, also prevent ETR1 signal output [4, 52]. Those studies suggest that although the RD and HK domain can be dispensable for ETR1 receptor signaling, both still have a role in the receptor signal output.

As mentioned, typical RRs/RDs undergo a conformational change in response to phosphorylation at the conserved Asp residue. There are structural similarities between inactive apo forms, but the phosphorylated, activated RRs/RDs are sometimes structurally diverse, probably because the active RRs/RDs can bind to various downstream proteins. With the phosphorylation deficiency, the ETR1-RD might already be in an active form in interacting with downstream components through different surfaces and in part convey the receptor function that may not necessarily involve the ethylene signaling. ETR1 ethylene receptor signaling could be independent of the RD. Our observation purposes the idea that ETR1-RD is an atypical type RD and has functional diversity.

## Materials and Methods

### The ETR1-RD constructs and site-directed mutagenesis

The cDNA fragment that encodes the receiver domain (RD) of *Arabidopsis thaliana* ETR1 (residues 605–738) was amplified by polymerase chain reaction (PCR) and cloned into the pET6H vector (modified from the pET11d vector, Novagen) with the N-terminal 10-residue His-tag M(H)_6_AMG [34]. The construct contains a total 144 residues with molecular weight of 16,228 Da. CheY-like ETR1-RD mutant containing 8 mutation sites (the red boxes in Fig 1) was prepared by site-directed mutagenesis, using the pET6H-ETR1-RD plasmid as the template. The constructed plasmids were verified by DNA sequencing.

### Expression, and purification of the recombinant proteins

The ETR1-RD and its mutant cDNA fragments cloned in pET6H were transformed into *E*. *coli* strain *BL21* (DE3) and grown in LB medium with 0.1 mg mL^-1^ ampicillin. The cells were cultured at 37°C until the OD_600_ reached 0.6 and were subsequently induced by adding isopropyl-1-thio-β, D-thiogalactopyranoside (IPTG) to a final concentration of 1 mM for 20 hours at 16°C. The cells were harvested by centrifugation, resuspended in lysis buffer (40 mM Tris-HCl, pH 8.0 and 150 mM NaCl), and lysed using a high-pressure homogenizer (GW technologies, Taiwan). The 6×His-tagged ETR1-RD was purified by affinity chromatography using Ni^+^-NTA agarose (GE Healthcare). The proteins were further purified by size-exclusion chromatography using a Sephacryl S-100 column (GE Healthcare) in FPLC buffer (40 mM Tris-HCl, pH 7.0, 150 mM NaCl and 1 mM EDTA). The purified proteins were verified by mass spectrometry. The protein concentration was estimated based on the extinction coefficient and absorption at 280 nm via Nanophotometer (IMPLEN). To prepare the ^15^N-labeled or ^15^N-, ^13^C-labeled samples for NMR, the cells were grown in M9 minimal medium supplemented with ^15^NH_4_Cl (1 g L^-1^) and ^13^C _D_-glucose (2 g L^-1^) as the sole nitrogen and carbon source.

### NMR titration experiments

Samples containing ~ 0.1 mM ETR1-RD were placed in NMR buffer (40 mM Tris-HCl, pH 7.0 and 150 mM NaCl in 90% H_2_O/10% D_2_O) to prepare them for NMR titration experiments. To study the phosphorylation of the ETR1-RD, the phosphoryl analog beryllofluoride (BeF_3_^-^) was used to mimic the phosphorylation process. BeF_3_^-^ readily forms a stable phosphoryl transfer complex and has been widely used to mimic aspartyl phosphate in the RDs of the TCSs. We prepared 1 M BeF_3_^-^ by mixing 1 M BeCl_2_ and 10 M NaF in NMR buffer. BeF_3_^-^ in 10 mM MgCl_2_, was titrated into the ETR1-RD solution to a final concentration of 3 mM. The BeF_3_^-^ induced chemical shift changes of the ETR1-RD were monitored by ^1^H-^15^N heteronuclear single quantum coherence spectroscopy (HSQC). The assignments of the ETR1-RD backbone N and NH resonances have been established in a previous study [34]. The ^1^H chemical shift was calibrated using 2,2-dimethyl-2-silapentane-5-sulfonate (DSS) at 0 ppm, and the ^15^N chemical shift was calibrated indirectly using DSS via their gyromagnetic ratios.

### NMR relaxation measurements

Measurements of the T_1_ and T_2_ relaxations and the [^1^H]-^15^N heteronuclear nuclear Overhauser effect (NOE) were performed at 25°C on a Bruker DRX600 spectrometer. T_1_ delays of 10, 50, 100, 200, 400, 600, 800, 1000, 1200 and 1500 ms were used with repeated 50, 400 and 1200 ms. T_2_ delays of 0, 17, 34, 51, 68, 102, 136, 170, 204 and 238 ms were used with repeated 34, 102 and 204 ms. For the [^1^H]-^15^N NOE measurement, independent saturated and unsaturated spectra were recorded in an interleaved manner. The spectral data were processed using NMRpipe [53]. The ^15^N T_1_ (= 1/R_1_) and T_2_ (= 1/R_2_) relaxation values were analyzed by fitting the series of peak intensities to an exponential decay curve in Sparky [54]. The NOE data were obtained by calculating the peak intensity ratios between the saturated and unsaturated NOE spectra. The relaxation parameters containing R_1_, R_2_ and NOE with error values were fitted to model-free equations using the Tensor2 program [55]. The rotational diffusion tensors were estimated using the X-ray monomer structure (PDB code 1DCF) as the template.

## Supporting Information

S1 Table The information of the 10 largest clusters of structure calculation. (DOC)
