# Supplementary material for: NMR Study Reveals the Receiver Domain of Arabidopsis ETHYLENE RESPONSE1 Ethylene Receptor as an Atypical Type Response Regulator
Source: PLoS One. 2016 Aug 3;11(8):e0160598. doi: 10.1371/journal.pone.0160598 (PMC4972365; doi:10.1371/journal.pone.0160598)
Supplement: S1 Table — (DOC) [file pone.0160598.s001.doc]

**Supporting Table S1**

**Table S1. The information of the 10 largest clusters of structure calculation.**

**
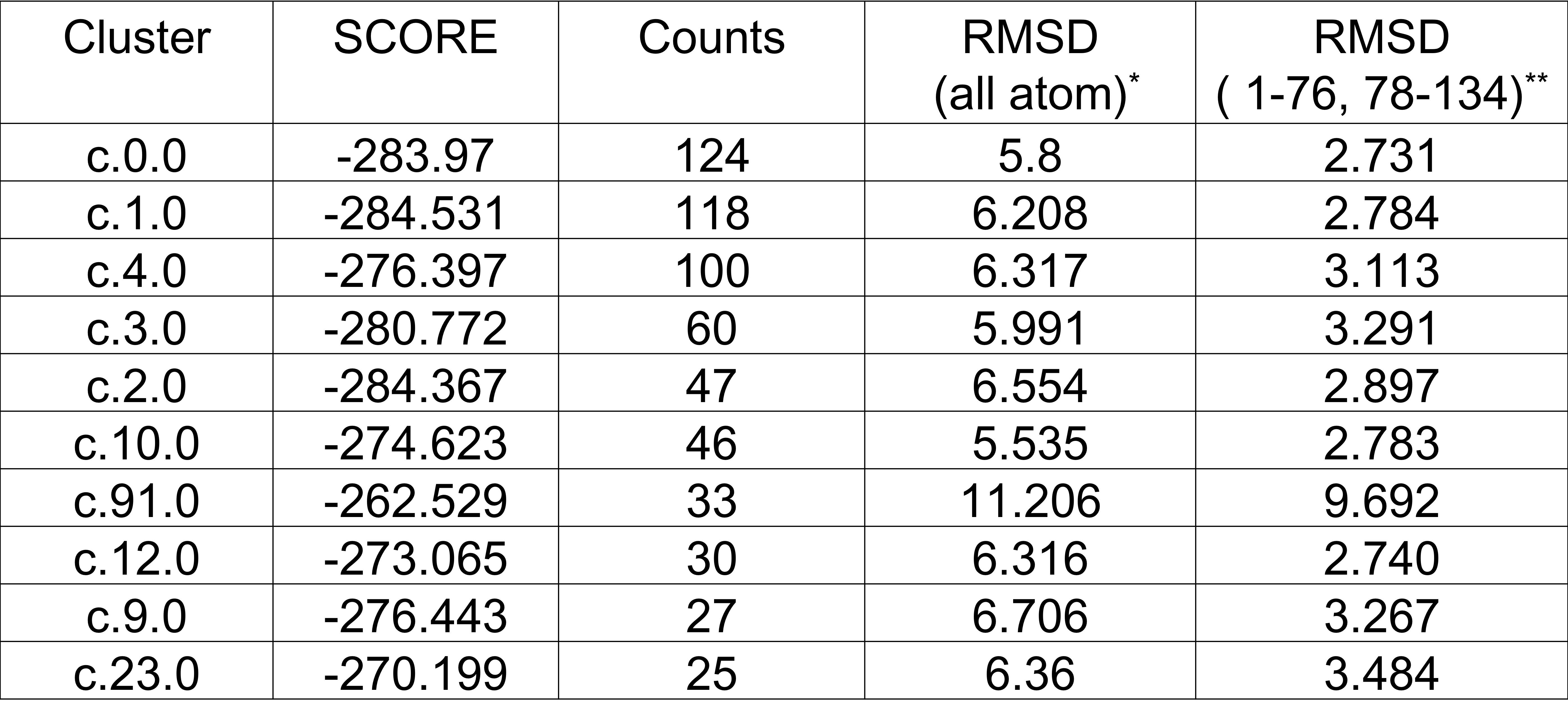
**

* Calculated by Rosetta program [1].

** Calculated by pymol scripts [2].

**References**

1. Shen Y, Vernon R, Baker D, Bax A. De novo protein structure generation from incomplete chemical shift assignments. J Biomol NMR. 2009;43(2):63-78.

2. Schrodinger, LLC. The PyMOL Molecular Graphics System, Version 1.8. 2015.
